# Geena 2, improved automated analysis of MALDI/TOF mass spectra

**DOI:** 10.1186/s12859-016-0911-2

**Published:** 2016-03-02

**Authors:** Paolo Romano, Aldo Profumo, Mattia Rocco, Rosa Mangerini, Fabio Ferri, Angelo Facchiano

**Affiliations:** IRCCS AOU San Martino IST, Genoa, Italy; Dipartimento di Scienza e Alta Tecnologia and To.Sca.Lab, Università dell’Insubria, Como, Italy; CNR - Istituto di Scienze dell’Alimentazione, Avellino, Italy

**Keywords:** Computational Proteomics, Computational Mass Spectrometry, MALDI/TOF mass spectrometry, MS spectra comparison/alignment, Automation of data analysis, Web application, LAMP

## Abstract

**Background:**

Mass spectrometry (MS) is producing high volumes of data supporting oncological sciences, especially for translational research. Most of related elaborations can be carried out by combining existing tools at different levels, but little is currently available for the automation of the fundamental steps.

For the analysis of MALDI/TOF spectra, a number of pre-processing steps are required, including joining of isotopic abundances for a given molecular species, normalization of signals against an internal standard, background noise removal, averaging multiple spectra from the same sample, and aligning spectra from different samples.

In this paper, we present Geena 2, a public software tool for the automated execution of these pre-processing steps for MALDI/TOF spectra.

**Results:**

Geena 2 has been developed in a Linux-Apache-MySQL-PHP web development environment, with scripts in PHP and Perl. Input and output are managed as simple formats that can be consumed by any database system and spreadsheet software. Input data may also be stored in a MySQL database. Processing methods are based on original heuristic algorithms which are introduced in the paper.

Three simple and intuitive web interfaces are available: the Standard Search Interface, which allows a complete control over all parameters, the Bright Search Interface, which leaves to the user the possibility to tune parameters for alignment of spectra, and the Quick Search Interface, which limits the number of parameters to a minimum by using default values for the majority of parameters.

Geena 2 has been utilized, in conjunction with a statistical analysis tool, in three published experimental works: a proteomic study on the effects of long-term cryopreservation on the low molecular weight fraction of serum proteome, and two retrospective serum proteomic studies, one on the risk of developing breat cancer in patients affected by gross cystic disease of the breast (GCDB) and the other for the identification of a predictor of breast cancer mortality following breast cancer surgery, whose results were validated by ELISA, a completely alternative method.

**Conclusions:**

Geena 2 is a public tool for the automated pre-processing of MS data originated by MALDI/TOF instruments, with a simple and intuitive web interface. It is now under active development for the inclusion of further filtering options and for the adoption of standard formats for MS spectra.

**Electronic supplementary material:**

The online version of this article (doi:10.1186/s12859-016-0911-2) contains supplementary material, which is available to authorized users.

## Background

Mass spectrometry (MS), a still fast improving technology, produces a high volume of data. Large interest therefore exists for MS data management and analysis, and tools are continuously developed to this aim.

In most cases, data are pre-processed at level of instrumentation (including both the instrument itself and the proprietary software that is distributed along with it) for noise subtraction, isotope abundance evaluation, range selection, normalization against a standard signal, and so on. Spectra are then saved for subsequent analysis.

However, the pre-processing is usually performed by applying different conditions, and adaptability of these steps would be useful. Moreover, human controlled re-application of pre-processing procedures on a large number of technical and/or experimental replicates becomes a recurring task, long and potentially source of errors [1].

It is also noteworthy that the analysis of the final spectrum can change depending on the experimental design. Indeed, when dealing with experimental replicates, it is of interest to generate an average spectrum, as recently demonstrated [2]. Furthermore, the spectra collected for different samples can be compared, in order to highlight similar signals or, on the contrary, dissimilarities between spectra.

The observation of common signals, or the search for distinguishing signals, in other words the search of biomarker signals, has been based on the comparison of mass spectra, and it has been used to classify patients’ samples [1, 3–5], to distinguish microorganisms and animal species [6–9], to detect specific neuropeptides [10]. As these studies demonstrate, the collection of large number of spectra, and their analysis by averaging of replicates and the comparison of the average spectra, is a common practice that represents a field of development for bioinformatics tools.

While many tools exist for MS data management and analysis, allowing most of these elaborations to be carried out by combining their use at different levels, little is currently available for the automation of the fundamental steps involved in the analysis of m/z and abundance data from MS experiments. In fact, it is often reported in literature, and in the previously cited studies, the practice of using single tools together with some manual steps of integration based on Excel data sheets or original scripts. The only alternative can be found by commercial packages, mostly distributed by the manufacturers of spectrometers. Therefore, we decided to develop a free, simple and dedicated MALDI/ TOF (Matrix Assisted Laser Desorption Ionization / Time Of Flight) MS data management and analysis tool, to be available to researchers by a web interface, able to provide an automatic pipeline of analysis of mass spectra, useful to compare them within proteomic studies on large numbers of samples.

### Aim of this work

For the analysis of MALDI/TOF spectra the following assumptions should be considered:

- in each spectrum, molecules may be represented by several signals, each corresponding to a different isotopic abundance: these can be summed up to give a total abundance value for each molecule;
- often, experimental data have to be normalized against an internal standard in order to obtain (semi) quantitative results;
- since experimental data are affected by background noise, the selection of signals above a threshold modulated on the spectra profile may be useful;
- the analysis of sample replicates yields multiple spectra which are different because of marginal errors/changes in the experimental phase only: an average spectrum representative of the sample may be determined by aligning these spectra along the m/z axis and computing mean intensity values from the corresponding abundances;
- the alignment along the m/z axis may be useful also for comparing spectra obtained from different samples, which could lead to the identification of significant differences in abundances and/or missing signals.

On the basis of these assumptions, we first developed Geena, a prototype tool for filtering, averaging and aligning MALDI/TOF spectra [11]. Geena main limitations are related to: i) the web interface, which is rather complex, since it includes all elaboration parameters in a single form, ii) the type and format of the output, which is also complex and difficult to re-use, and iii) the performances, which are especially poor when the number of spectra involved in the analysis is great.

We present here Geena 2, a redesigned, revised, more performing and much more user-friendly version of Geena.

## Methods

### Work and data flow of the system

**Fig. 1:**
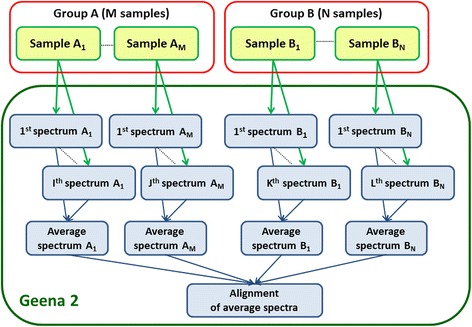
Geena 2 work and data flow. The final alignment is achieved by aligning the average spectra which are computed by averaging all spectra from the same sample. Samples may be grouped according to some common origin or characteristics, *e.g.* cases and controls. Reference to the original spectra is maintained in the final alignment, and this can therefore be passed to other tools for further analysis, *e.g.* for differential analysis and biomarker discovery

Geena 2 work and data flow are summarized in Fig. 1.

In differential proteomics, it is common that two or more groups of samples are compared in order to identify differences, *e.g.*, case samples (patients affected by a given pathology) against control samples (unaffected persons). For each of these groups, a variable number of samples can be made available. In other situations, grouping of samples is not available *a priori*, and a classification of the samples is instead investigated.

In both cases, a variable number of spectra (replicates) is generated for each sample. This is where the automatic procedure implemented by Geena can be usefully adopted. Replicates may be pre-processed, as described in the following Algorithms sub-section, and “averaged”, thus producing an “average spectrum” which is representative of the sample. Finally, these average spectra can be aligned. The alignment is the basis for any subsequent analysis, *e.g.* case–control comparison, classification, or biomarker discovery, according to data provided in input.

### Development environment

We developed and made available Geena 2 by using the Linux-Apache-MySQL-PHP (LAMP) web development framework, which is an easy to manage, effective working environment, on a cloud based virtual server, currently with a configuration consisting in a quad core AMD64 2 GHz CPU with 7 Gb RAM memory. The operating system presently is Ubuntu Server 14.04.3 LTS. Current Apache, PHP and MySQL versions respectively are 2.4.7, 5.5.9 and 5.5.47, which are provided by the Ubuntu distribution.

All code originally written for Geena has been revised, corrected and partially rewritten for sake of performances and because of changes introduced in the interfaces, as well as in the output format. Geena 2 is written in PHP [12], but for the spectra alignment which is instead written in Perl [13]. In more detail, the alignment of spectra is performed with a local run of the NEAPOLIS tool [14], which uses an original procedure to compare spectra, based on the sorting of all m/z values from spectra under analysis, and the alignment of the values included within an user-defined range of m/z. The aligned signals are then used to generate an average spectrum, and mean and standard deviation are computed for m/z and intensity values. Details about the alignment procedure are available at the NEAPOLIS web site.

### Data format

Input spectra are managed as tab or comma separated values (respectively, TSV and CSV) text files. These formats can easily be produced by the majority of spectrometers associated software and consumed by any database system and spreadsheet software. For the analysis of the same data with variable parameters, input data may also be stored on our server in a MySQL [15] database and reused, although this feature is only presently available upon collaboration with the developers. The format of the input is described below, while the Additional file 1 includes a full example input file that can be used for testing purposes.

**Fig. 2:**
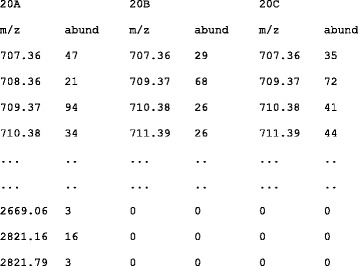
Text excerpt: Input file example. Here only the first and last peaks of three spectra from the same sample are shown. Values are separated by tab characters. Since the spectra may have a variable number of peaks, missing values are replaced by "0"s

The input is composed of blocks, each including information on spectra generated from the same biological sample. Values of mass over charge and of related abundance for each spectrum are reported in column. The first spectrum is on columns 1 and 2, the second on columns 3 and 4, and, in general, the n-th spectrum occupies columns n*2–1 for the m/z value and n*2 for the related abundance. Since spectra usually have a variable number of signals, the number of rows corresponds to the highest number of signals in all spectra. Missing values in shorter spectra are replaced by “0”s. The first row includes labels describing the spectra. The second row includes the headers for the data columns, usually the texts “m/z” and “abund”, replicated as many times as the number of spectra available for the sample. The text excerpt in Fig. 2 includes a simple example showing three spectra from the same sample, as previously described. Each block is separated by the next one by a row that includes only two back slash characters, *i.e.* “\\”, at the begin of the row.

The number of output files is considerably higher than in Geena. Output spectra are provided as TSV/CSV files. Additional output is provided as HTML files. In Geena 2, for each spectrum under analysis the output includes:

- the original spectrum, as a list of peak pairs representing the m/z and abundance values of the peak, named the “Original” file,
- the filtered spectrum, as a list of peak pairs as above, including only peaks selected after the pre-processing analysis, named the “Filtered” file,
- information on grouping of isotopic replicas, including lists of selected peaks, each with the list of peak pairs from the original spectrum that were grouped together, named the “Isotopic groups” file.

For each group of replicate spectra from the same sample, Geena 2 output also includes the “average spectrum”, achieved by aligning and averaging all filtered spectra from the same sample, together with related alignment information, which are provided both as text file, for further processing, and as HTML file, for better readability. The average spectrum includes (m/z, abundance) pairs which are computed as average of respective m/z and abundance values for equivalent signals found in the filtered spectra.

**Fig. 3:**
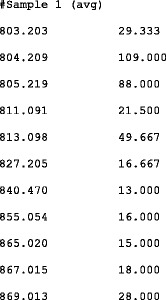
Text excerpt: Average spectra example. Only the first rows are shown. Here, m/z and abundance values are computed by averaging the corresponding values of averaged spectra. The peak selection process is reported in the manuscript

**Fig. 4:**
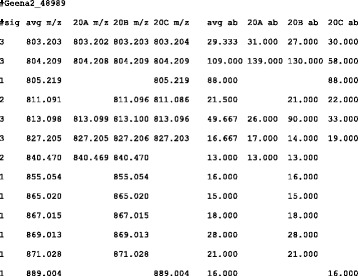
Text excerpt: Alignment example. Only the first rows are shown. This alignment was derived from three average spectra. In the first column, the number of spectra involved in the determination of each peak is reported. The following four columns report m/z values (average and single spectra). The last four columns report the abundances (average and single spectra). For better readability, an HTML version of the file is also available

The short text excerpt in Fig. 3 includes a simple example showing an average spectrum, as previously described. Similarly, the text excerpt in Fig. 4 includes a simple example showing alignment information as text file for an average spectrum generated by aligning three spectra from the same sample formatted as previously shown in Fig. 2.

In this file, which is meant to be passed on to other software tools for further analysis, values are separated by a tabulation character, and this can limit its readability. Users should better see the equivalent HTML file. The first column reports the number of spectra that were found to include the given peak. From the second to the fifth column, m/z values are shown, the first value being the average one. Empty values correspond to missing peaks in the corresponding spectrum. From the sixth to the last column, abundance values are reported with the same format of m/z values.

### Algorithms

The processing methods are based on original heuristic algorithms.

They include:

- pre-processing of spectra replicates, which in turn consists in:
  i) isotopic peaks identification and joining: signal peaks which are separated by 1 m/z unit (Dalton) in mono-charged species may represent the same molecules, with different isotopic composition, if and only if their abundance values follow the expected distribution, which is variable according to the m/z values of involved peaks. For low mass peptides, the mono-isotopic species largely is more represented than others. At greater masses, non mono-isotopic species gain a greater relevance, having peaks which may become higher than the mono-isotopic one. While the m/z value at which the second species is higher than the first one can only be determined when the exact peptide composition is known, we have experimentally verified by using the Molecular Weight Calculator software [16] that this usually happens for peptides of molecular weight higher than 1,700 Da. When at least two peaks are recognized as representative of the same molecular species, their abundances are summed up and assigned to the first peak in the series, the monoisotopic one, while the other peaks are removed. Two parameters, namely “Maximum number of isotopic peaks” and “Maximum delta between isotopic peaks”, are available to make this identification and joining tuneable according to the instrument used for generating the spectra. Joining information is provided as output, for verification, in HTML;
  ii) normalization: a simple normalization of abundance values is performed by assuming 100.00 the value of a given normalization peak, if available, in order to improve the estimate of the abundances and making them comparable in different acquisitions;
  iii) peak selection: a threshold line can be built and only signal peaks above it are selected. The line is built by defining some reference threshold values at given m/z values, including the lowest and highest m/z values for the spectra, and by linearly interpolating between them. Up to six reference values can presently be defined, thus allowing creating a threshold line able to adapt to the shape of the spectra under analysis.
- computing average spectra for replicates: spectra which are generated by analysing the same sample can be “averaged” in order to remove single analysis artefacts and strengthen the quality and representativeness of the resulting spectrum.
  For this, “equivalent” signals, that is signals that are supposed to represent the same molecular species in original spectra, are first identified by selecting the nearest peaks among those having a difference in m/z values below a given threshold parameter (“Maximum delta for aligning replicates”). If equivalents peaks for a signal are present for a number of spectra which is higher than a given parameter (“Minimum number of signals in replicates”), then both the m/z and abundance average values are computed and assigned to a peak of the average spectrum.
- alignment of average spectra: average spectra which are generated by averaging replicated spectra from the same sample, can be aligned in order to highlight differences in signals among samples.
  In this case too, equivalent signals, that are supposed to represent the same molecular species in average spectra, are first identified by selecting the nearest peaks among those having a difference in m/z values below a given threshold parameter (“Maximum delta for aligning average spectra”). If equivalents peaks for a signal are present for a number of spectra which is higher than a given parameter (“Minimum number of signals in average spectra”), then both the m/z and abundance average values are computed and assigned to a peak of the aligned spectrum.

## Results

### Web interfaces

Contrary to its predecessor, Geena 2 is available on-line for public access through three simple and intuitive web interfaces. The Standard Search Interface (SSI) allows the user a complete control over all parameters and it is of special interest for expert users and for users with special needs. A simplified interface, named Bright Search Interface (BSI) is also available. It allows users to control both average and alignment parameters, while filter parameters are taken by default. Finally, the Quick Search Interface (QSI) allows users to run Geena 2 by using only a few parameters: the analysis range in m/z and the mass of the normalization peak, if applicable. In this case, default values are used for the majority of parameters.

**Fig. 5:**
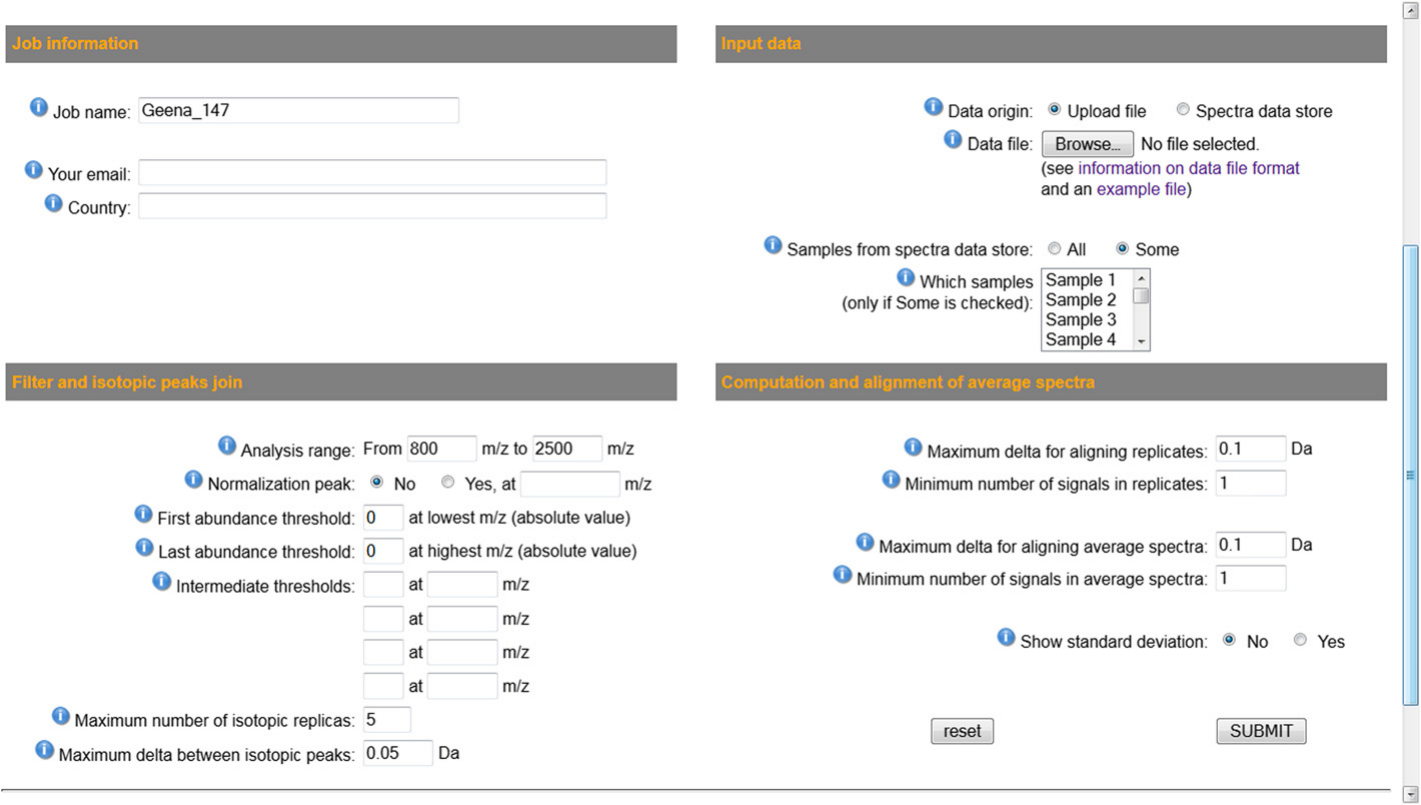
The Standard Search Interface (SSI). The Standard Search Interface (SSI) includes four sections. The majority of parameters have an associated default value, which can be changed. By default, the normalization peak is not taken into account and the threshold filtering is not used

The SSI includes four input sections, as it is shown in Fig. 5. The first section (“Job information”) asks for optional information as a job name, an email for sending results, and country of work for statistics aims.

The second section (“Input data”) is meant to define data input method and to specify data to be analysed. Two methods are available: file upload and selection of data from a local database. The second option is only available for collaborating parties. It may be especially useful for repeated analysis with variable parameters. The third section (“Filter and isotopic peaks join”) allows specifying in detail the requested pre-processing tasks and the related parameters. It allows defining the analysis range in Dalton by defining minimum and maximum m/z values to be taken into account. It also supports the definition of the presence of a normalization peak and of its m/z value in Da. The linear filter, when desired, is also specified in this section by introducing the threshold values at various m/z. Finally, the parameters related to the optional joining of isotopic peaks can also be specified in this section.

The fourth section (“Computation and alignment of average spectra“) allows to set the parameters for averaging the replicates and for generating a final alignment and an average spectrum for all spectra under analysis.

**Fig. 6:**
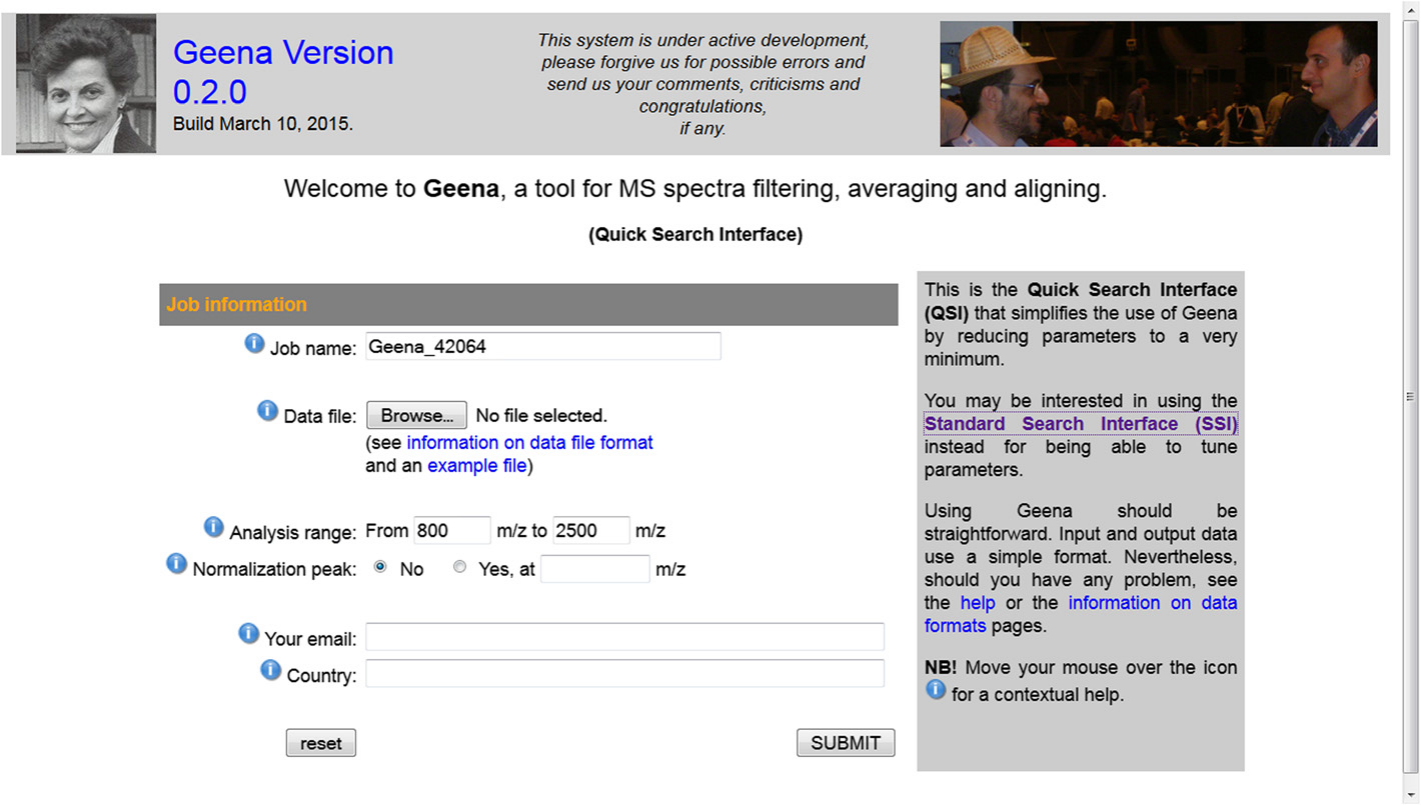
The Quick Search Interface (QSI). The Quick Search Interface (QSI) only includes essential parameters, which cannot be assumed by default: the input file, the analysis range and the presence of a normalization peak. Default values are assumed for all remaining parameters

The QSI (see Fig. 6) assumes that the majority of parameters involved in the analysis may have assigned a default value and it therefore only includes a reduced number of input parameters. Moreover, it does not allow selecting the input data from the local database.

**Fig. 7:**
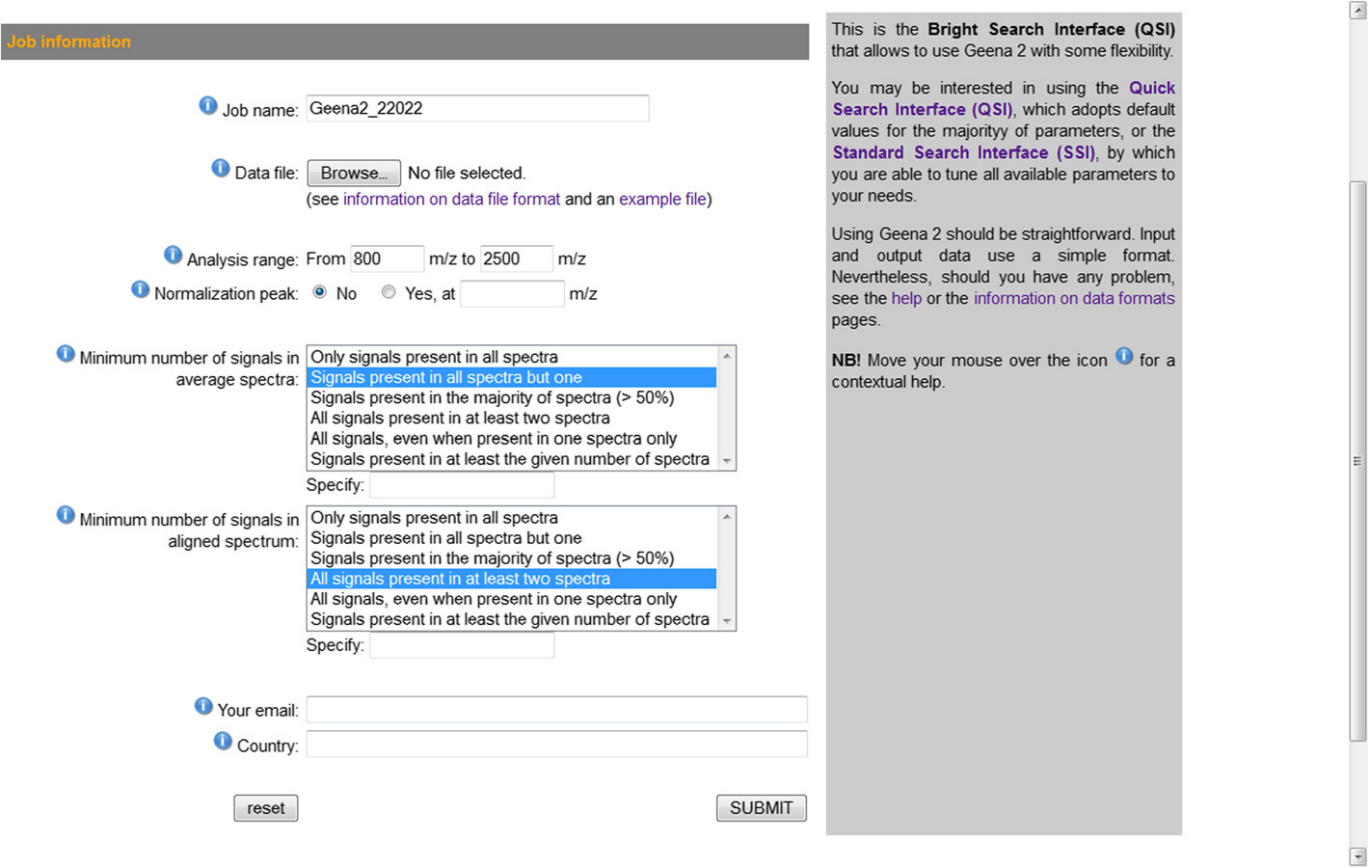
The Bright Search Interface (BSI). In respect to the QSI, the Bright Search Interface (BSI) includes additional parameters able to tune the averaging and alignment processes. A high number of signals is preferable for the averaging process, while a low one is better for the alignment process. Default values are assumed for all remaining parameters

The BSI (see Fig. 7) allows users a greater control over the averaging and alignment processes. As the QSI, it assumes that the majority of parameters have assigned a default value and only allows to upload data from a file. Additionally, it allows users to specify, both for the averaging and for the alignment processes, the number of spectra, among those being aligned, that must present a given signal for this be included in the average or alignment. Possible options in this case are to select:

- Only signals present in all spectra
- Signals present in all spectra but one
- Signals present in the majority of spectra (>50 %)
- All signals present in at least two spectra
- All signals, even when present in one spectra only
- Signals present in at least a number of spectra specified by the user

A high number of spectra presenting the signal is preferable for the averaging process, which determines the average spectrum of spectra replicates for the same sample. In this case, only those signals that are present in many, if not all, spectra is included in the average spectrum, thus avoiding to include sporadic signals, possibly arising from the spectrum acquisition process. On the contrary, a low number of signals is better than a high one for the alignment process, where average spectra from distinct samples are compared. In this case, signals are included in the alignment even when they are rare, thus facilitating and empowering the following differential analysis.

**Fig. 8:**
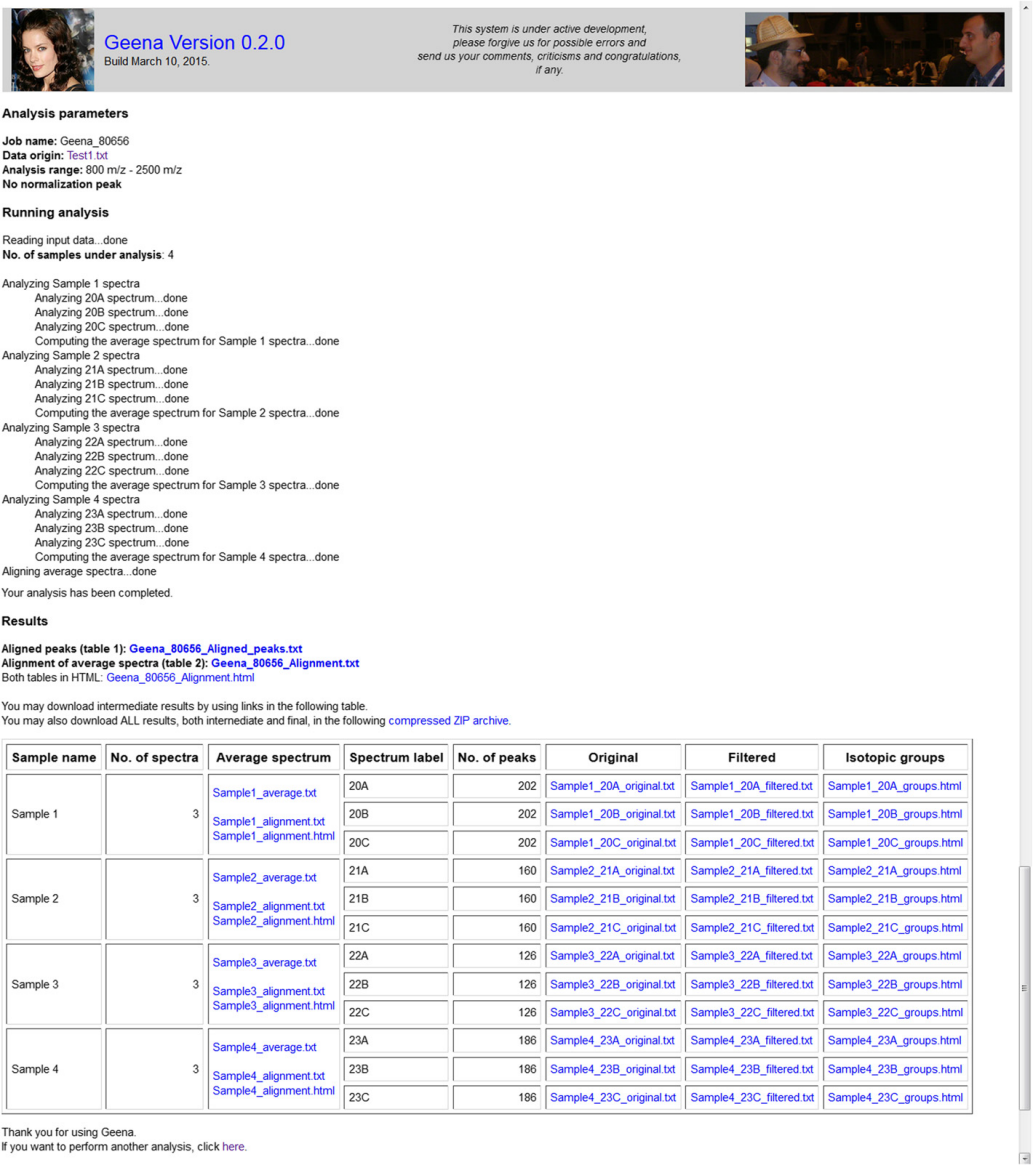
The output page. The upper part of the output page includes the parameters used for the analysis, according to the search interface used. It also includes, in the “Running analysis” section, a list of analysis carried out by Geena 2: this is compiled and progressively shown at run time, so that the user knows which analysis is being performed at any time. The lower part of the output page (“Results” section) includes results of the analysis, both intermediate and final. Intermediate results are shown in a table for better readability. Each result in included in a separate file which is linked by a self-explaining text and file name. All results may also be downloaded in a compressed archive

The main output consists, as specified in the Methods section and shown in Fig. 8, in the alignment of all average spectra, which were in turn built by aligning and averaging all replicated spectra for each given sample. This is provided in a variety of formats: aligned spectrum in TSV format (list of signals resulting from the alignment with the relative average abundance computed from abundances of peaks in the average spectra that contributed to the peak in the alignment), complete alignment in TSV format (including the average spectrum together with m/z and abundance values of all signals in the average spectra), complete alignments in HTML.

Intermediate outputs contain filtered MS spectra and their alignments. All results are available for downloading from the result page and a link to a compressed archive is also available in the results page and sent by email.

## Discussion

### Validation and application examples

In the following, we present three examples of experiments where the analysis of MS spectra generated with a MALDI/TOF instrument was carried out by using either Geena 2 or its previous releases.

First, we carried out a proteomic study on the effects of long-term cryopreservation on the low molecular weight fraction of serum proteome (3). Blood samples used in this study either were stored at −20 °C for 8 years after previously being collected from 17 healthy donors or were freshly collected from 13 other unrelated healthy donors. From the freshly collected samples, two sets of aliquots were generated. One set was stored at −80 °C and analysed 18 months later, whereas eight samples of the second set were immediately subjected to analysis. Raw data generated by MALDI/TOF analysis within a dynamic m/z range from 800 to 2,500, were exported as an Excel spreadsheet. Data handling was performed using “PROTEO” and “NEAPOLIS”, two bioinformatic tools that were later merged and formed Geena. PROTEO allowed isotopic peaks joining, normalization, and peak selection, while NEAPOLIS was used both to obtain the mean abundance values for experimental replicates and to align mass spectra along the m/z axis. Statistical analysis performed on data elaborated by NEAPOLIS generated a panel of 106 m/z values whose abundances were significantly different between the experimental groups.

Geena was for the first time employed in a retrospective proteomic study on cryopreserved sera from patients affected by gross cystic disease of the breast (GCDB), a common benign disease of the mammary gland, affecting some 7 % of women in Western countries [4]. For the realization of this study two experimental groups were established: a) thirty women who developed a breast cancer following first cyst aspiration served as cases, b) sixty women who, by the same date limit, were still breast cancer free served as controls. Each sample was analysed in quadruplicate by MALDI/TOF MS. Raw data were pre-processed by Geena which generated a panel of 96 candidate signals to be subjected to statistical analysis. The following Significance Analysis of Microarray (SAM), performed by taking as input the results provided by Geena, identified a significant increase in serum levels of complement fraction C3f in GCDB patients who during the follow-up developed a breast cancer.

Recently, the combined utilization of Geena 2 and SAM allowed the identification of angiotensin II serum level as a predictor of breast cancer mortality following breast cancer surgery [17]. In this case, an independent validation of Geena 2 data processing has been obtained by using the alternative bio-analytical technique ELISA immunoassay, which confirmed the same results.

Despite the fact that in many applications the peak selection can be achieved by means of the Geena 2 linear filter described in the previous section, noisy datasets might demand a more accurate treatment. In the works reported above an alternative external filtering tool, a stand-alone National Instruments LabVIEW [18] program named MS-BASELINER was used. MS-BASELINER is able to dynamically evaluate data background along the spectrum, discriminating between spurious, noisy peaks and real peaks, through an iterative process which determines a background noise value for each peak in the spectrum by analysing all peaks around it only. MS-BASELINER can be downloaded from the Geena 2 website along with a detailed description [19].

### Comparison with existing tools

Despite the existence of many bioinformatic tools covering the main aspects of proteomics investigation, the lack of online tools suitable for creating an archive of MALDI MS spectra and to analyse-reanalyse them with different parameters makes Geena unique under this aspect. Concerning the single steps of analysis performed, there are tools available for pre-processing of MS spectra within the MatLab bioinformatics tool box as well as in R, two development environments largely used by bioinformatics, but, in our experience, not simple to use for biochemists and MS analysts. On the other hand, tools for aligning spectra are available within the same MatLab and R environments, as well as in form of independent tools [20, 21]. The unique feature of Geena 2, however, is its workflow architecture, that makes it possible to integrate other tools for each step of the analysis, leaving into the interface the opportunity to select the most suitable to the user work. As further development, the integration of freely available tools for the pre-processing, filtering, and aligning of spectra will be considered to make the tool even more flexible and useful to a wider community.

## Conclusions

Elaborating from previous tools PROTEO, NEAPOLIS, and Geena, the original software Geena 2 can now be considered a useful public-domain, free tool for the automated pre-processing of relatively uncomplicated MS data, like those originated by MALDI/TOF instruments. Its flexible, yet simple and intuitive web interface, as well as the simple layout of the results, makes it very powerful, yet easy to use even for not particularly experienced operators. Its output can straightforward be used as input for further analysis, including differential analysis and biomarker discovery, by using many publicly available software tools.

It has proven to be useful and effective for published scientific works, where it has been adopted for pre-processing of MALDI/TOF spectra which were then compared by using statistical software on a limited amount of data.

The tool is publicly available on-line and it can also be distributed on collaborations.

### Future directions

Current developments of Geena 2 include an extension of its pre-processing, filtering, and aligning features and of accepted input and output formats.

As discussed, the MS-BASELINER filter has been adopted for a more sophisticated identification of background noise. A new version of this tool is being implemented within Geena 2 and will be optionally available soon. Other filtering methods are under evaluation. Since mzML [22] is now a standard format for archiving and exchanging MS spectra, Geena 2 will soon adopt it for input data.

Further developments under evaluation include parallelization of alignment tasks and the creation of user-specific environments accessible after user authentication allowing both the upload of confidential data in a controlled access table of the database and the definition of preferred parameters for processing and alignment of spectra.

## Availability and requirements

Project name: Geena 2

Project home page: http://bioinformatics.hsanmartino.it/geena2/

Operating system(s): Ubuntu 14.04.3 LTS

Programming languages: LAMP Framework, PHP and Perl

Other requirements: Apache HTTPD, MySQL

License: no license defined, software available on collaborations

Any restrictions to use by non-academics: none

## Additional file

Additional file 1:
**An example input file for Geena 2.** This example file can be used for testing purposes. It includes 12 MS spectra generated by MALDI/TOF from four biological samples in the context of a real experiment. Three spectra were generated for each sample. The format of the file is described in details, and with examples, in the manuscript and in the information file on Input/Output data formats in the web site. (TXT 26 kb)

## Abbreviations

AMD: advanced micro devices
BSI: bright search interface
CPU: central processing unit
CSV: comma separated values
GCDB: gross cystic disease of the breast
HTML: HyperText Markup Language
LAMP: Linux-Apache-MySQL-PHP
m/z: mass-to-charge ratio
MALDI/TOF: matrix assisted laser desorption ionization time-of-flight
MS: mass spectrometry
mzML: mass spectrometry markup language
PHP: PHP: Hypertext Preprocessor (recursive definition, originally “Personal Home Page”)
QSI: quick search interface
RAM: random access memory
SAM: significance analysis of microarray
SSI: standard search interface
TSV: tab separated values
